# Characteristics of Microcellular Foamed Ceramic Urethane

**DOI:** 10.3390/polym13111817

**Published:** 2021-05-31

**Authors:** Jin Hong, Soo-hyun Cho, Chang-Seok Yun, Dong Hwi Kim, Youngjae Ryu, Sung Woon Cha, Yong Hoon Jang

**Affiliations:** 1 School of Mechanical Engineering, Yonsei University, 50, Yonsei-ro, Seodaemoon-gu, Seoul 03722, Korea; jin.hong@yonsei.ac.kr (J.H.); donghwi.kim@yonsei.ac.kr (D.H.K.); yjryu1027@yonsei.ac.kr (Y.R.); 2 Lens Manufacturing Technology Group, Module Solution (Biz Unit), Samsung Electro-Mechanics, 150, Maeyeong-ro, Yeongtong-gu, Suwon-si 16674, Gyeonggi-do, Korea; sh0116.cho@samsung.com; 3 New Business Intiative Team, NPC, 129, Idang-Ro, Gunpo-si 15853, Gyeonggi-do, Korea; csyun@npc.co.kr

**Keywords:** microcellular foam, batch process, ceramic urethane, composite, polymer

## Abstract

Ceramics are non-metallic inorganic materials fabricated from natural or high-purity raw materials through heating and cooling processes. Urethane is a three-dimensional plastic with both elasticity and chemical resistance; moreover, it is used as a rubber substitute. The use of both materials in various applications is gradually increasing. However, as ceramics and urethane have distinctly different properties, this prompted questions regarding the properties of a material that is fabricated using both materials. Therefore, we studied the characteristics of a composite material fabricated through physical foaming using a batch process. The process was conducted with gas saturation, foaming, cooling, and curing. When a specimen of 2 mm thickness was saturated in 5 MPa of CO_2_ for 2 h, the solubility was 6.43%; when foaming was carried out at a temperature of 150 °C in boiled glycerin, the foaming ratio, cell size, cell density, and void fraction were found to be 43.62%, 24.40 µm, 9.1 × 10⁷ cells/cm^2^, and 22.11%, respectively. Furthermore, the volume increased by 102.96%, color changed from dark to light gray, hardness decreased by 24%, thermal diffusivity increased by 0.046 mm^2^/s at 175 °C, and friction coefficient decreased to 0.203. Thus, the microcellular foamed ceramic urethane exhibits a larger volume, lighter weight, and improved thermal conductivity and friction coefficient.

## 1. Introduction

The material properties of solids are divided into six categories: mechanical, thermal, magnetic, optical, electrical properties, and corrosion resistance. In engineering materials, processing and performance factors are also indispensable. Since the structure changes depending on how the material is processed, it greatly influences the performance of the final product. Many researchers characterize according to the material properties so that one of the thousands of available materials can be selected for the suitable material. However, the material is rarely perfectly ideal for use. Like the relationship between stiffness and ductility, a moderate compromise must be made [1].

Industrial materials include metals, alloys, polymers, ceramics, glass, composite materials, and natural materials, with over 50,000 types of materials in total. Metals and metal alloys have been predominantly used as industrial materials; however, polymeric and composite materials have gradually garnered attention globally. The choice of materials is a part of the design process. Most mechanics, such as dynamics and statics, have already established a theory or principle, and making them change is difficult. However, it is possible to achieve a new theory or principle that develops new properties of the material. Therefore, the designer’s focus should be on the characteristics of the material, not the material itself. The inherent mechanical properties of the material influence the product design and account for the most significant proportion of the cost of manufacturing the final product. The introduction of new or multiple process methods is essential for solving the shortage of industrial materials that may soon occur, particularly considering environmental factors for waste generated by using these materials (Table 1) [2,3].

Ceramic and urethane are used in various practical applications; research and development on them are being actively conducted. The proposed microcellular foamed polymer improves insulating properties owing to the decrease in conductivity and is suitable for various acoustic fields because of the attenuation of the cell/matrix interface. In addition, the polymer has improved thermal and mechanical properties and reduced cost because of an expansion in volume [4]. Ceramics are used in various applications such as biomedical materials, metal-ceramic composites, high specific strength materials, and products using absorbents and filtration catalysts. Several types of foaming methods are being developed, and ceramics that aid in the fabrication of micro-sized cells through foaming are garnering technical attention. Thus, the market has seen rapid development in this regard; moreover, the versatility of ceramic lies in its endless potential.

Therefore, it was necessary to study the properties of a new composite by mixing urethane, which is typically low in density and has good flexibility, and ceramic, with excellent heat and electrical performance and high strength. Although these two materials are very different in material properties, they are readily available around us and have in common that they are already used in many places.

To maximize the advantages of ceramic and urethane, the types of frequently used functional materials have been studied extensively. In particular, because it is a material that can achieve various combinations, repeated attempts to study topics such as blending ratio and method, foaming conditions, and foaming methods are being conducted. With continued research, ceramics, and urethanes have become invaluable in our lives [5,6,7,8].

However, the needs of customers are gradually diversifying because of the rapidly changing environment, lifestyles, and social culture development. Discussions on materials capable of satisfying customers are actively taking place. Furthermore, meeting the requirements of both the necessary specifications and the diversity of product types are emphasized. Additionally, environmental damage and human health play important roles. Owing to rapid industrialization and the use of plastics and freon gas, the planet is experiencing abnormal changes in climate, and various species of animals and plants are under threat. To combat this, the government of each country is preparing various systems and tools that can be legally managed; it has become an essential issue for the consideration of developers and users. Furthermore, the characteristics of a material synthesized using ceramic and urethane has not yet been studied in detail. Therefore, research and development of such a composite material must proceed with the observation of the changed characteristics after its synthesis.

Efficient design of a relatively inexpensive material that incorporates the beneficial characteristics of extant materials is therefore necessary. We conducted a study on how ceramic and urethane cross-linked sheets with completely different physical properties are changed through the batch process, an eco-friendly microcellular foaming method, and the physical changes and improvements in properties gained after the process.

## 2. Materials and Methods

**Figure 1:**
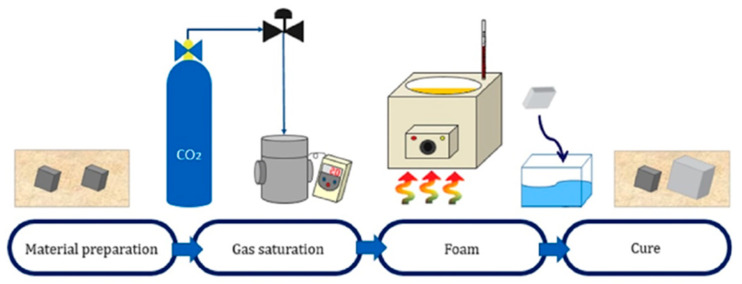
Procedure of the batch process.

The batch process (Figure 1), a foaming method developed by Martini in 1979, enables microcellular foaming in a simple manner. This process can achieve improved results at identical strength, fracture toughness, and insulation properties with the use of less material. The fabrication of microcellular foam varies drastically in terms of cell generation, depending on the saturating gas type, gas pressure, solubility, foaming temperature, and foaming time [9].

### 2.1. Materials

In this study, a ceramic urethane sheet (MISUMI Group Inc., Seoul, Korea, Product code No. UTSCM) was purchased. The specimens were cross-linked after mixing the ceramic powder with urethane comprising polyester polyol. The specimens were 25 mm wide, 25 mm long, and 2 mm thick. The mechanical properties of the specimens were as follows: a specific gravity of 1.25~1.28, shore hardness of A 70, heat resistance of 70 °C, cold resistance of −20 °C, tensile strength of 53 MPa, and elongation of 680%. Based on the elemental analysis results (PerkinElmer Inc., Seoul, Korea, Product no. 2400 Series II CHNS/O), the fabricated composite consisted of 55.86% carbon, 7.72% hydrogen, 5.06% nitrogen, 0.60% sulfur, and other components.

This ceramic urethane sheet is manufactured by mixing urethane and granulated ceramic powder to maximize abrasion resistance. The average size of the ceramic particles was 2.04 μm, and maximum size was 13.07 μm, and the minimum size was 0.42 μm. The particle distribution included about 14.22% of large-sized particles (more than 5 μm), 71.56% of medium-sized particles (less than 5 μm to more than 1 μm), and about 14.22% of small-sized particles (less than 1 μm). It was checked that the ceramic powder exhibiting the above distribution occupied 2% of the total specimen.

### 2.2. Equipment

A batch process was used to fabricate the microcellular foam. The specimens were saturated with carbon dioxide (Samheung, Seoul, Korea; product grade no. CO_2_) in a vessel with an inner diameter of 52 mm and a height of 200 mm, which was equipped with an electric heater. In the foaming phase, an oil bath (Chang Shin Science Co., Seoul, Korea, Product No. C-WHT) and 99.50% glycerin were used to produce microcellular-sized cells. An electronic densimeter (Alfa Mirage, Model No. MD-300S) and electronic scale (OHAUS, Model no. AR2130) was used to measure the densities and weights of the specimens.

### 2.3. Microcellular Foam Processing Method of Ceramic Urethane

Satisfactory solubility is an important condition for microcellular foaming. To predict this condition, information on the thickness of the material used and the diffusion coefficient of the saturation gas are required.

**Figure 2:**
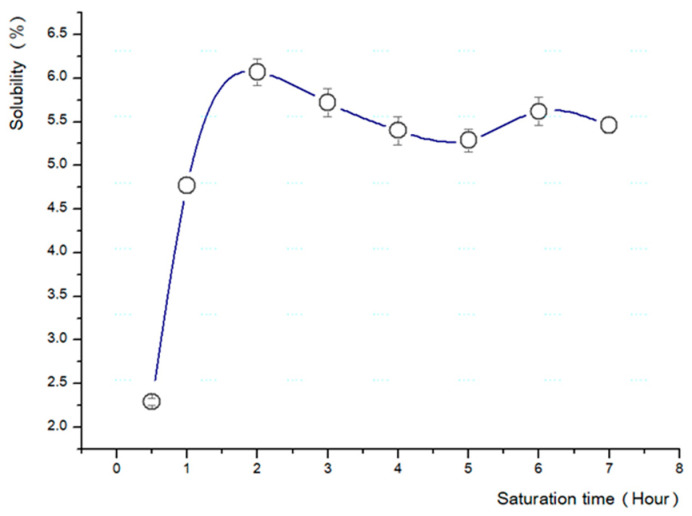
Changes in solubility corresponding to saturation time.

Carbon dioxide, a supercritical fluid, was selected as the saturated gas on behalf of the ethylene, ethane, dinitrogen monoxide, isopropanol, water, toluene, propane, ammonia, and more, since it is non-flammable, non-toxic, inexpensive, and has a near-ambient critical temperature. Carbon dioxide can be described as a hydrophobic solvent with polarity comparable to that of n-hexane. Hence, nonpolar or light molecules easily dissolve in supercritical carbon dioxide, whereas the polar or heavy molecules have very poor solubilities. Some light organic compounds, either polar or nonpolar, are used as co-solvents to enhance carbon dioxide’s solvating power and polarity. It is the most representative supercritical fluid, and it dissolves various substances very well. It has been industrially used in various processes, including polymerization, polymer fractionation, particle formation for pharmaceutical and military use, textile dyeing, and cleaning of machines and electronic parts. Moreover, it is one of the most commonly used gases for microcellular foaming. Therefore, we used carbon dioxide for saturation. [10] The diffusion coefficients of carbon dioxide are $1.0\times 10^{-5}$ cm^2^/s and $1.0\times 10^{-6}$ cm^2^/s (at 250 bar and 353 K) [11]. The saturation time was calculated using the following equation:

(1) $$t\;≅\;0.55\;L^{2}/D$$

where *L* is the thickness of the specimen and *D* is the diffusion coefficient of the gas. Since a specimen of 2 mm thickness and carbon dioxide were used in this experiment, the saturation time, *t*, was calculated to be a minimum of 2200 s to a maximum of 22,000 s. Based on these data, saturation was checked every 1 h from a minimum of 30 min to a maximum of 7 h at 60 °C of vessel temperature in absolutely the same way as the batch process (Figure 2) [12].

The prepared specimens were placed in a gas injection vessel. Four specimens were placed in one vessel, where a space between the specimen and another specimen must be made for optimized saturation of the gas. It was directly related to the foaming ratio. Therefore, we wrapped the specimens with a paper towel and made a gap between them so that the specimens did not touch each other. We confirmed that the lid replaced with the new rubber packing was tightly sealed. The specimen was removed from the vessel after saturating it in 5 MPa carbon dioxide for 2 h. The weights of the specimens were immediately measured, and the initial and post-saturation weights of the specimens were determined. A total of five methods were available to measure the solubility; among them, we employed the gravimetric method [13] (pp. 5–10). The solubility of the specimens was determined using the following equation:

(2) $$\mathrm{Solubility}\;(\%)=\frac{{\mathrm{Weight}\;}^{\mathrm{Gas}\;\mathrm{out}}-{\;\mathrm{Weight}\;}^{\mathrm{Gas}\;\mathrm{in}}}{{\mathrm{Weight}}^{\;\mathrm{Gas}\;\mathrm{in}}}\;\times \;100$$

**Figure 3:**
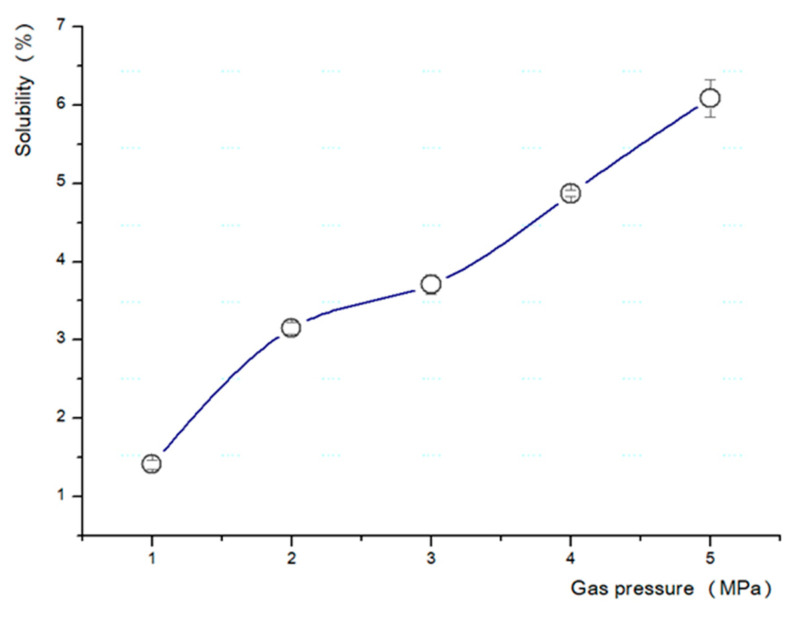
Changes in solubility corresponding to saturation gas pressure.

**Figure 4:**
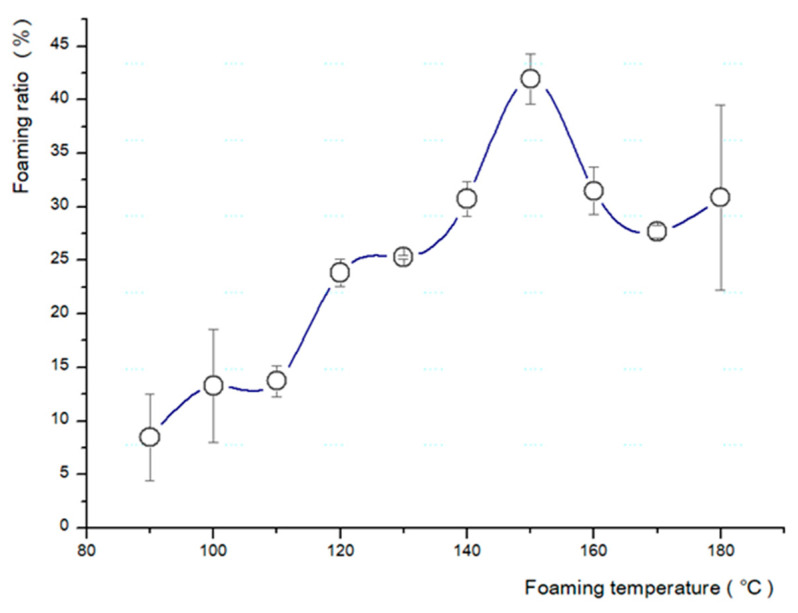
Changes in foaming ratio corresponding to foaming temperature.

Based on the above results, a test was conducted to determine the most suitable temperature and pressure for the fabrication of the microcellular foam (Table 2). The pressure was increased by 1 MPa (from 1 to 5 MPa) (Figure 3), and the temperature of the boiling glycerin was increased by 10 °C (from 90 to 180 °C) (Figure 4).

The specimens were fully saturated by carbon dioxide foamed by imbalanced heat conditions. Subsequently, as pores form in the material, the density of the specimen decreases. The foaming ratio is a value consisting of the cell size and number of cells of a microcellular foamed plastic, which is demonstrated by measuring densities before and after foaming [14]. The specimens were subsequently placed in an oil bath containing boiled glycerin, and foam processing was performed. The time required was approximately 60 s; when the gas saturation was low, the process required a longer duration, and when the saturation was high, the process required a relatively short duration. The density measured using ASTM D792-20 was calculated as the foaming ratio using the following equation [15,16]:

(3) $$\mathrm{Foaming}\;\mathrm{ratio}\;(\%)=\frac{{\mathrm{Density}\;}^{\mathrm{Before}}-{\;\mathrm{Density}\;}^{\mathrm{After}}}{{\mathrm{Density}\;}^{\mathrm{Before}}}\;\times \;100$$

After the foaming process, curing was performed for approximately one week until no change in the foaming ratio and shrinkage was observed in all foamed specimens.

**Figure 5:**
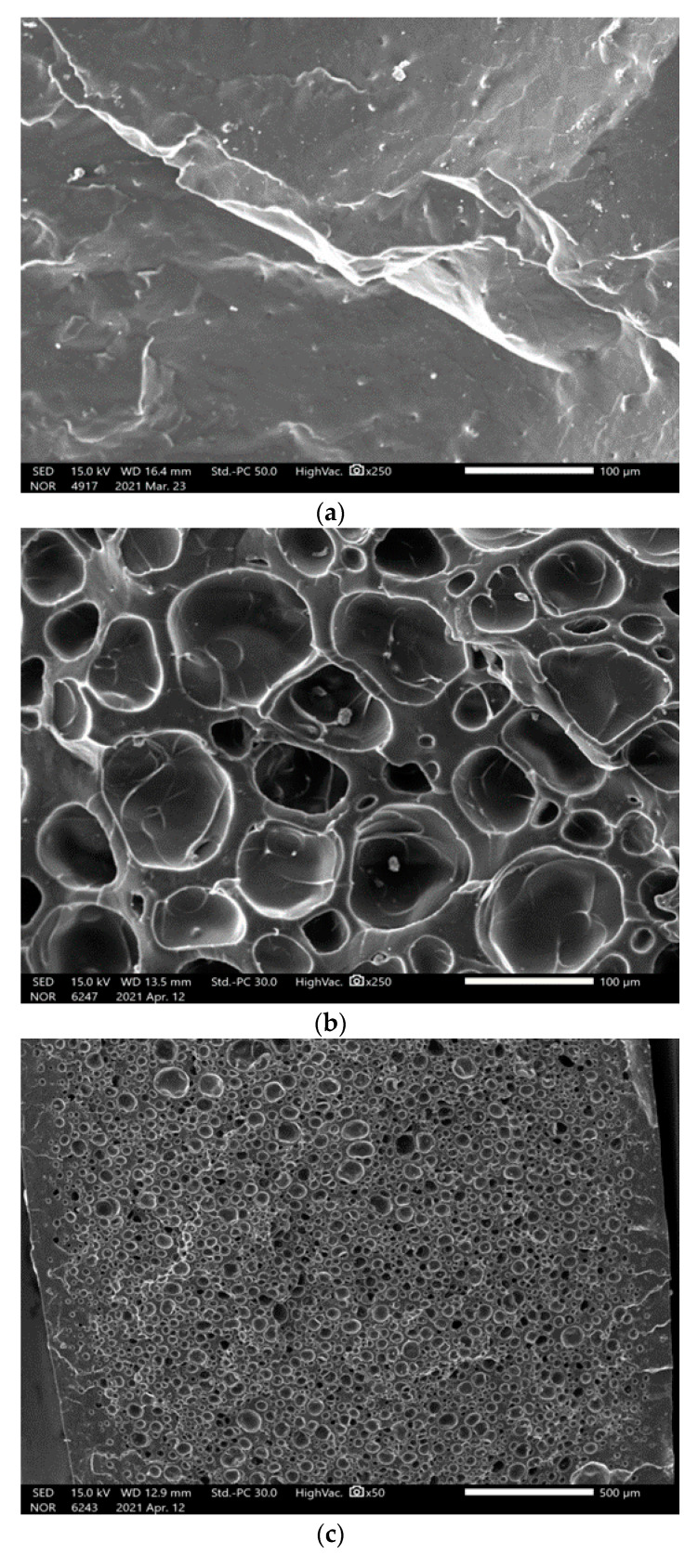

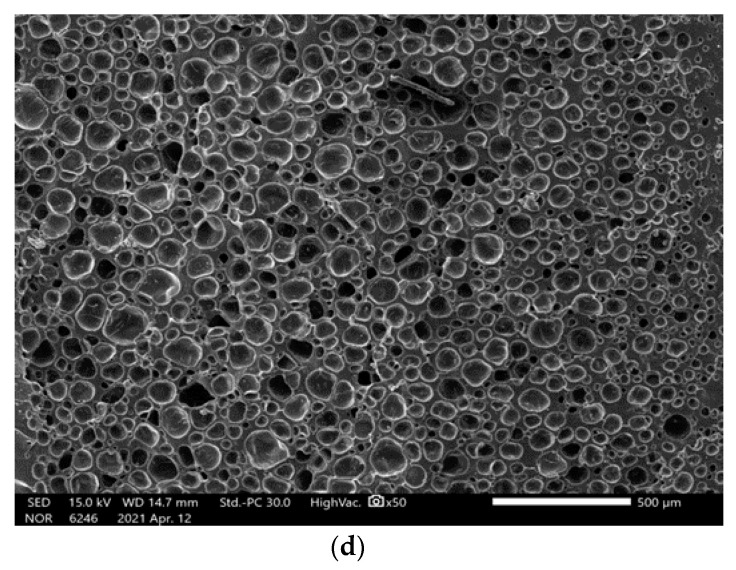
Scanning electron micrograph of ceramic urethane specimens: (**a**) Original (at a magnification of ×250) (**b**) Microcellular foamed at a temperature of 150 °C (at a magnification of ×250) (**c**) Microcellular foamed at a temperature of 130 °C (at a magnification of ×50); (**d**) Microcellular foamed at a temperature of 150 °C (at a magnification of ×50).

The expansion of the appearance can recognize the cell growth of the microcellular foam and can be accurately confirmed using scanning electron microscopy (SEM; JEOL Ltd., Massachusetts, USA, FE-SEM Model no. IT-500). To conduct an SEM analysis, we froze the foamed specimens using liquid nitrogen (supplied by Samheung, Seoul, Korea) and subsequently broke and pretreated them to aid clear visibility of the cross-section. The processed specimens were photographed after plasma processing (Cressington Scientific Instruments Ltd., Watford, UK, Sputter coaters Model no. Cressington 108 auto) for approximately 120 s [17]. As shown in Figure 5, cells (micro-sized cells) that did not exist before foaming were created during the cellular foaming process. Cell size was measured using ImageJ, and the following equation expresses the relationship between the cell size, density, and void fraction:

(4) $$V_{f}=\left(\frac{\pi }{6}\right)d^{3}N_{f}$$

and,

(5) $$N_{0}=\frac{N_{f}}{\left(1-V_{f}\right)}$$

where $N_{0}$ is the cell density of the foamed specimen, *d* is the cell size, and $V_{f}$ is the void fraction [12].

## 3. Results

The types of saturation gas, pressure, solubility, foaming time, and foaming temperature directly influence the fabrication of the microcellular foam [4]. We observed that 2 h was the optimal saturation time for a 2 mm specimen, the solubility was approximately 6.43%, the saturation gas pressure was 5 MPa, and the ideal foaming temperature was 150 °C for the base conditions.

### 3.1. Cell Growth and Changes in External Shape

The foaming ratio achieved was 43.62%, the average cell size was found to be 24.40 µm, the cell density was 9.1 × 10⁷ cells/cm^2^, and the void fraction was 22.11% (Table 3).

### 3.2. Altered Characteristics of Microcellular Foamed Ceramic Urethane

#### 3.2.1. Shape and Hardness

**Figure 6:**
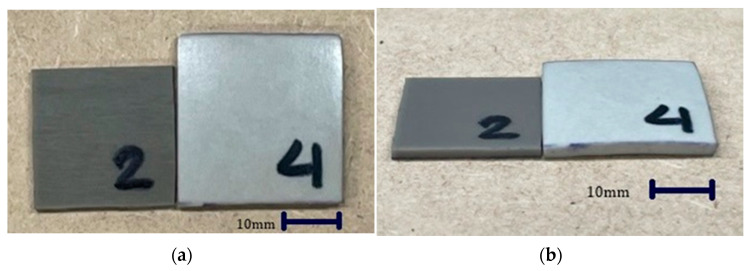
Changes in the volume and color of the specimens: (**a**) Top view (**b**) Side view.

An alteration in the shape and color of the microcellular foamed ceramic urethane was clearly visible (Figure 6). These changes can be determined by measuring the weight, density, and thickness of the specimens.

**Figure 7:**
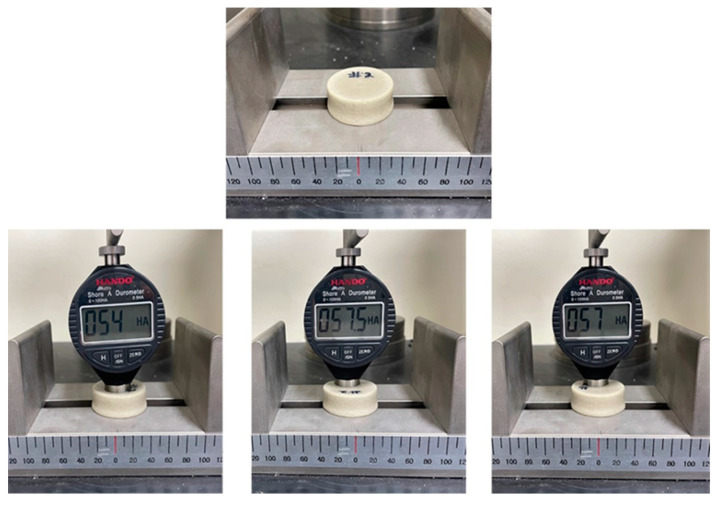
Shore A hardness value of the microcellular foamed ceramic urethane.

The volume of the specimen increased by 102.97% and the color changed from dark to light gray; however, the specimen that had a higher foaming ratio turned brighter. Shore A hardness was measured using a digital hardness tester (HANDO-Midyo, Korea, Shore A hardness tester, Model No. HD-KR10A). The hardness of the non-foamed ceramic urethane was 70, and that of the microcellular foamed ceramic urethane was 56 (Figure 7), that is, the hardness decreased by approximately 24.5% [18].

#### 3.2.2. Thermal Diffusivity

The changes in the thermal diffusivity of the ceramic urethane specimens were analyzed using LFA 457 (MicroFlash^®^, NETZSCH Korea Co., Ltd., Paju, Korea, Laser flash apparatus Model no. LFA 457). The measurements were performed starting from a reference temperature of 25 °C to a maximum temperature of 200 °C. Additionally, measurements were made at intervals of 25 °C, and nitrogen was used as the gas.

**Figure 8:**
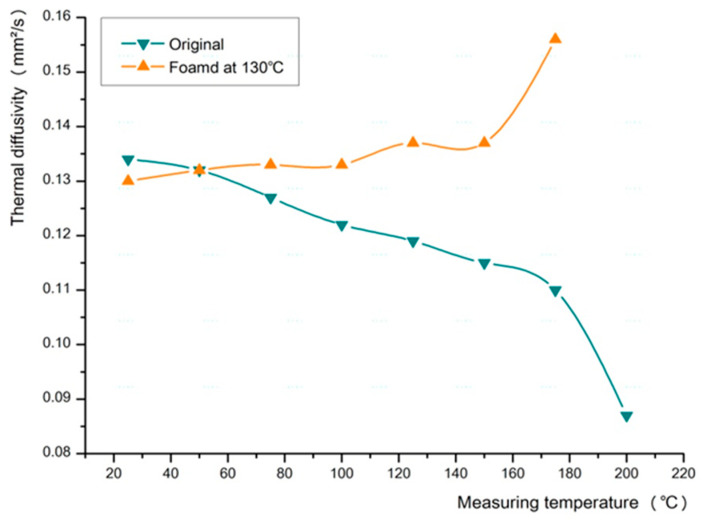
Changes in the thermal diffusivity of ceramic urethane.

When the two materials of different thermal properties were mixed, the thermal characteristics of the fabricated material were similar to those of urethane. We observed that the thermal diffusivity of the original specimen gradually decreased as the measurement temperature increased, whereas the thermal diffusivity of the foamed specimen increased as the measurement temperature increased (Figure 8). Thus, we confirmed that ceramic urethane exhibits improved thermal diffusivity through microcellular foam processing [19,20].

#### 3.2.3. Coefficient of Friction

**Figure 9:**
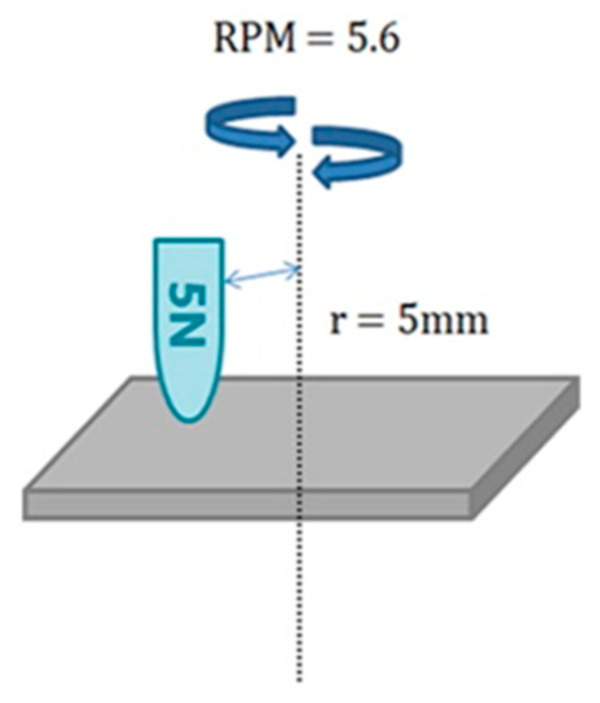
Conditions set for the tribometer.

The change in the friction coefficient of the ceramic urethane sheets was measured using a tribometer (Anton Paar Korea Ltd., Seoul, Korea, Pin-on-Disk tribometer Model no. TRB^3^). The test conditions were as follows: 1 mm alumina milling media balls, rotation radius was 5 mm, vertical load was 5 N, and RPM was 5.6, and the results were confirmed after 180 cycles (Figure 9) [21].

**Figure 10:**
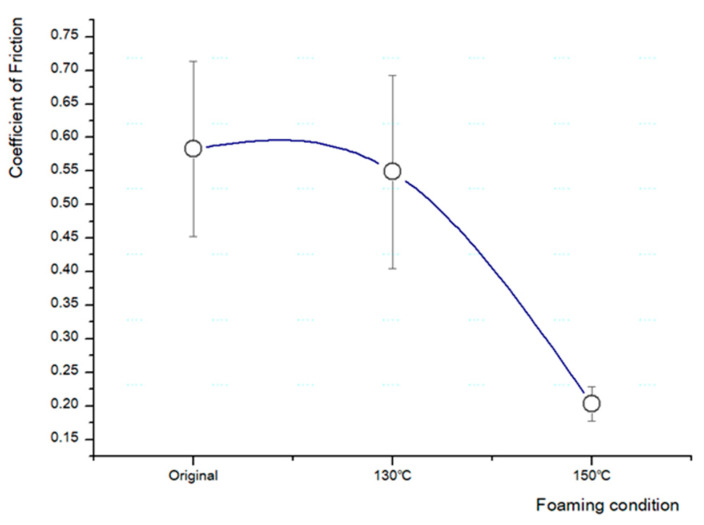
Changes in the friction coefficient of ceramic urethane.

The coefficient of friction of the original sheet was 0.583, which gradually decreased when the foaming temperature was increased. After foaming at 150 °C, a total decrease from 0.38 to 0.203 was observed, that is, the coefficient of friction approximately halved after foaming (Figure 10).

When the surfaces in contact move relative to each other, the friction between them causes the kinetic energy to be converted into thermal energy. In addition, this property manifests as another critical result, leading to poor performance or component damage. This can be prevented in two ways: by coating the surface to strengthen or reduce friction [2,22,23].

## 4. Discussion

Ceramic and urethane are materials with different properties. By treating these two materials as a single material, we fabricated a composite material comprising micro-cells using a batch process. Parameters such as the types of saturation gas, saturation pressure, saturation temperature, foaming time, and temperature directly influence the microcellular foam. Therefore, we studied and derived the ideal value for foaming ceramic urethane through experiments and finally obtained a microcellular foamed ceramic urethane material.

The importance of optimization and systematization of microcellular foaming conditions based on the construction method was established in this study. Even if identical ceramic urethane sheets were to be used, the optimal usage would depend on the size, shape, and density of the cell obtained through the microcellular foaming process. It was possible to obtain the mechanical and thermal properties that could be compared with rubber or metal by using the existing standard; however, it was difficult to confirm the inherent properties of ceramic urethane. Lastly, we attempted to discover new characteristics of the fabricated material by employing various techniques during the microcellular foaming process.

A composite material fabricated by mixing two or more materials is obtained by integrating the best characteristics of each constituent material and by achieving a combination of properties that are not exhibited by any single material. Ceramic and urethane have advantages and disadvantages on opposite sides; however, it is worth developing ceramic urethane characteristics by controlling methods and various conditions.

Ceramic and urethane are currently very easily found around us and are useful materials in many places. In terms of price and safety, they are also excellent. However, since this microcellular foamed ceramic urethane material that we have developed has never been used before and has different characteristics than those found in a single product, it is necessary to find a place where the microcellular foamed ceramic urethane sheet can be applied in terms of engineering. Currently, applications using ceramic urethane are deficient. These include construction materials [24], dendrite-free solid-state batteries [25], insulation paints, and waterproof paints, and more. Therefore, we have a plan to research microcellular foamed ceramic urethane of soundproofing and absorption sound. [26] The physical properties of the material, which are further expanded after foaming, can be beneficial and can also reduce manufacturing costs by increasing the material’s properties with fewer processing steps and time. In addition, once a suitable application for this material is found, and we are confident of its remarkable performance. Moreover, its infinite capacity will continue to be identified and confirmed through future research.

## 5. Conclusions

We succeeded in fabricating microcellular foam using a batch process; the fabricated material, which comprises ceramic and urethane, is harmless to humans and the environment. Various studies and experiments have been conducted. The specimens were saturated with carbon dioxide at a pressure of 5 MPa for 2 h at 60 °C and then physically foamed in a glycerin bath at 150 °C.

We achieved the following results from the experiment: the solubility, foaming ratio, cell size, cell density, and void fraction were found to be 6.43%, 43.62%, 24.40 µm, 9.1 × 10⁷ cells/cm^2^, and 22.11%, respectively. Furthermore, the volume increased by 102.97%, the color changed from dark to light gray, the hardness decreased by 24%, the thermal diffusivity increased by 0.046 mm^2^/s at 175 °C, and the friction coefficient decreased by approximately half to 0.203.

According to the results, synthesis using the microcellular foaming process results in synthesized material that has a larger volume, lighter weight, improved thermal conductivity, and lower friction coefficient. In other words, it exhibits the positive characteristics of ceramic and urethane. However, it is necessary to study other characteristics, such as vibration and soundproofing, in the further study of this material.

## Figures and Tables

**Table 1:** Material Properties.

Property	Ceramic	Urethane
Softening/melting point (K)	2323	365
Yield strength (${\mathrm{MN}}_{m}{}^{-2}$)	5000	26–31
Tensile strength (${\mathrm{MN}}_{m}{}^{-2}$)	250–550	58
Young’s modulus (${\mathrm{GN}}_{m}{}^{-2}$)	385–392	2.6–3
Stiffness (${\mathrm{GN}}_{m}{}^{-2}$)	100	2
Ductility (%EL)	-	2–5.5
Specific heat (J/g℃)	0.8	1.9
Thermal conductivity (W/m K)	16–29	0.12–0.18
Ionization energy ($\mathrm{kJ}/{\mathrm{mol}}^{-1}{\;\mathrm{of}\;O}_{2}$)	Large & Positive	≈−400

**Table 2:** Experimental conditions.

Microcellular Foam—Batch Process	
Saturation gas	Carbon dioxide
Saturation press. (MPa)	5
Saturation time (h)	2
Saturation temperature (°C)	60
Foaming fluid	Glycerin
Foaming time (s)	60
Foaming temperature (°C)	150
Experimental temperature (°C)	20 ± 3
Humidity (RH %)	65

**Table 3:** Results of the fabrication of microcellular foamed ceramic urethane.

Material	Ceramic Urethane
Solubility (%)	6.43
Density (g/cm^3^)	0.709
Foaming ratio (%)	43.62
Average of cell size (µm)	24.40
Cell density (cell/cm^3^)	9.1 × 10⁷
Void fraction (%)	22.11

## Data Availability

The data presented in this study are available upon request from the first author.
